# Immunotherapy and vaccination against infectious diseases

**DOI:** 10.1007/s00508-020-01746-2

**Published:** 2020-12-16

**Authors:** Meinolf Ebbers, Christoph J. Hemmer, Brigitte Müller-Hilke, Emil C. Reisinger

**Affiliations:** 1 Department of Tropical Medicine and Infectious Diseases, Rostock University Medical Center, Ernst-Heydemann-Str. 6, 18057 Rostock, Germany; 2 Core Facility for Cell Sorting and Cell Analysis, Rostock University Medical Center, Schillingallee 70, 18057 Rostock, Germany

**Keywords:** Infectious disease, Immunotherapy, Immunomodulation, Bacterial infections, Monoclonal antibodies

## Abstract

Due to the overuse of antibiotics, infections, in particular those caused by multidrug-resistant bacteria, are becoming more and more frequent. Despite the worldwide introduction of antibiotic therapy, vaccines and constant improvements in hygiene, the burden of multidrug-resistant bacterial infections is increasing and is expected to rise in the future. The development of monoclonal therapeutic antibodies and specific immunomodulatory drugs represent new treatment options in the fight against infectious diseases. This article provides a brief overview of recent advances in immunomodulatory therapy and other strategies in the treatment of infectious disease.

Immunization against smallpox was being carried out in Asia by means of variolation as early as around 1700. The exudate from smallpox blisters was introduced into the nose of the person being inoculated with the help of long bamboo canes [1, 2]. At the end of the eighteenth century the British country doctor Edward Jenner introduced vaccination against smallpox, replacing the far more dangerous method of inoculation. Finally, in 1979 the World Health Organization (WHO) announced the worldwide eradication of smallpox. The first vaccine against typhoid fever was developed in 1886, the first vaccine against cholera in 1896 and the first vaccine against the plague in 1897. The first vaccines against *Bordetella pertussis* followed in 1926 and the Bacille Calmette-Guérin (BCG) vaccine against tuberculosis in 1927. In the years after 1970 vaccines against *Streptococcus pneumoniae, Neisseria meningitidis, Haemophilus influenzae type B*, anthrax and cholera were developed. A vaccine against borreliosis was approved by the US Food and Drug Administration (FDA) in 1998, but it was withdrawn from the market within less than 2 years due to more than 1000 reports of side effects [3]. The development of new vaccines has the potential to reduce not only infectious disease morbidity and mortality, but also the use of antibiotics and, consequently, the development of antimicrobial resistance. Recently, a recombinant vaccine against two of the most common serotypes K1 and K2 of the hazardous bacterium *Klebsiella pneumoniae* has been successfully utilized to protect mice against fatal infections. *Klebsiella* are common pathogens of nosocomial urinary and respiratory tract infections, and particularly antibiotic-resistant types, such as carbapenemase-bearing or extended spectrum beta-lactamase (ESBL) positive *Klebsiella* strains are characterized by complex and sometimes challenging therapeutic approaches [4]. Besides antimicrobial resistance, vaccines also have the potential to reduce morbidity and mortality in acute global healthcare crises and to reduce the transmission of infectious diseases in our globalized world. This is especially relevant regarding the ongoing pandemic of the novel severe acute respiratory syndrome coronavirus 2 (SARS-CoV-2). The virus is a single-stranded RNA virus causing life-threatening respiratory illnesses [5]. According to the US Center for Systems Science and Engineering (CSSE) until 12 May 2020 the virus has infected approximately 4.2 million people and caused 286,000 deaths [6]. To end the coronavirus disease 2019 (COVID-19) pandemic, the world is in urgent need of an effective, safe and durable vaccine. Currently at least 8 candidate vaccines are being tested in clinical trials and 102 candidate vaccines are in preclinical evaluation. Most of the candidate vaccines are mRNA or DNA vaccines, live vaccines, vector virus vaccines, inactivated vaccines or viral protein subunits vaccines. First results from human trials are expected within the next few months [7]. Since SARS-CoV‑2 also affects pets and other animals, an animal vaccine is also urgently needed [8].

Another vaccine urgently needed is against *Plasmodium falciparium*. According to the World Malaria Report 2019 an estimated 228 million cases and 405,000 deaths of malaria occurred worldwide in 2018. Several promising vaccine candidates are currently under investigation. As of 18 May 2020, there are 199 registered trials involving malaria vaccines [9]. Currently, RTS,S/AS01 is thought to be the most advanced and extensively tested candidate vaccine against *P. falciparum.* The subunit vaccine induces immune response against the *P. falciparum* circumsporozoite protein (PfCSP) [10]. Although it is currently the only vaccine showing reliable efficacy against *P. falciparum *in a phase III clinical trial, its protective properties are only partial and decline over time [10, 11]. Despite these limitations, it is thought to exhibit public health benefits, especially in children in areas with high transmission and disease activity rates [12]. Unfortunately, funding volume levels for vaccine research and development decreased from US $ 181 million in 2017 to US $ 156 million in 2018, which may have dramatic effects on the development of malaria vaccine research in the future [13].

Not only have new vaccines been discovered in recent decades, numerous vaccines have also been significantly improved in terms of tolerability, safety and efficacy. One example is the vaccination against pertussis, which was discovered around 1930. At the time, the inactivated bacteria *B. pertussis* was administered for vaccination purposes; however, the inactivated whole cell vaccine frequently produced side effects in the form of redness and swelling at the injection site accompanied by fever and agitation. In the 1980s the whole cell vaccine was replaced by the acellular pertussis vaccine, which had fewer side effects. Currently, the inactivated pertussis toxin is used as an antigen in the production of the acellular vaccine, either alone or in combination with other cell components [14]. The central element in the development of highly selective vaccines is the identification of a specific and potent antigen structure that induces a long-lasting immunity preventing infection. By inducing a highly selective immune response, cross-reactions and side effects can be reduced. Lipopeptide, lipopolysaccharide and nucleotide-associated adjuvants target toll-like receptors among other receptors, while genetically modified helper cell epitopes support dendritic cells in their recognition of foreign antigens. Simultaneously, immunostimulatory peptides further enhance the immune response [15].

Vector-based vaccines belong to a category of a new subtype of vaccines in which genes from viruses or bacteria which code for structures that trigger a neutralizing immune response are introduced into apathogenic carrier viruses (e.g. measles or yellow fever viruses). One example of a carrier virus is the measles strain Schwarz. The carrier virus itself acts as an adjuvant-like initiator. Consequently, no conventional adjuvant is required. The structural genes introduced multiply with and within the carrier virus in the vaccinated person, thus inducing a neutralizing immune response. Since these gene segments can be specifically selected and inserted, immunogenicity and side effects can be predicted and assessed relatively well [16, 17]; however, one concern that has been discussed is the possibility of a reduced immune response in subjects with pre-existing immunity due to previous measles vaccinations or measles disease in the past [18]. The fear is that existing and persistent antibody titres against the measles virus could prevent the replication of the carrier virus; however, this only seems to play a minor role in humans since adequate immune responses were detected after vaccination with a measles virus into which a chikungunya antigen had been introduced even in individuals with high pre-existing measles titres [19]. Therefore, viral vectors make the development of various antibacterial vaccines, e.g. against tuberculosis, seem feasible in the future.

At the beginning of the twentieth century, Emil von Behring, Paul Ehrlich and Shibasaburo Kitasato managed to develop hyperimmune sera, which functioned as antitoxins against diphtheria and tetanus toxins, thus establishing a treatment option known as serum therapy [20]. Both von Behring and Ehrlich were awarded the Nobel Prize in Physiology or Medicine for their research, von Behring in 1901 for his work on serum therapy and Ehrlich in 1908 for his work on immunity (Table 1). During the same period other approaches to the prophylaxis and treatment of infectious diseases were also explored. Later, in the 1970s hyperimmune sera largely came to be replaced by monoclonal antibodies (mAb). From the 1980s onwards, the acquired immune deficiency syndrome (AIDS) epidemic prompted the search for immune modulators that may influence and modulate specific immune functions, the aim being, of course, to bring about an improvement in immune functionality [21]. Since then, numerous new modulators, antibiotics, antivirals, monoclonal antibodies and vaccines have been developed for the treatment and prophylaxis of infectious diseases.

**Table 1:** Overview of Nobel laureates and their scientific contributions to infectious disease and infection immunology research. Between 1901 and 2019, the Nobel Prize was awarded 109 times to 219 scientists. Only 12 women received the Nobel Prize in Physiology or Medicine. The mean age of prize winners was 58 years, while the youngest awardee was 32 and the oldest 87 at the time of the award ceremony. Source: The Nobel Prize, https://www.nobelprize.org/prizes/lists/all-nobel-laureates-in-physiology-or-medicine/

Year	Nobel Laureates	Prize motivation
1901	Emil Adolf von Behring	*For his work on serum therapy, especially its application against diphtheria, by which he has opened a new road in the domain of medical science and thereby placed a victorious weapon against illness and deaths in the hands of the physician*
1902	Ronald Ross	*For his work on malaria, by which he has shown how it enters the organism and thereby has laid the foundation for successful research on this disease and methods of combating it*
1905	Robert Koch	*For his investigations and discoveries in relation to tuberculosis*
1907	Charles Louis Alphonse Laveran	*In recognition of his work on the role played by protozoa in causing diseases*
1908	Ilya Ilyich Mechnikov, Paul Ehrlich	*In recognition of their work on immunity*
1919	Jules Bordet	*For his discoveries relating to immunity*
1926	Johannes Andreas Grib Fibiger	*For his discovery of the Spiroptera carcinoma*
1927	Julius Wagner-Jauregg	*For his discovery of the therapeutic value of malaria inoculation in the treatment of dementia paralytica *(syphilis)
1928	Charles Jules Henri Nicolle	*For his work on typhus*
1939	Gerhard Domagk	*For the discovery of the antibacterial effects of prontosil*
1945	Sir Alexander Fleming, Ernst Boris Chain, Sir Howard Walter Florey	*For the discovery of penicillin and its curative effect in various infectious diseases*
1948	Paul Hermann Müller	*For his discovery of the high efficiency of DDT (dichlorodiphenyltrichloroethane) as a contact poison against several arthropods*
1951	Max Theiler	*For his discoveries concerning yellow fever and how to combat it*
1952	Selman Abraham Waksman	*For his discovery of streptomycin, the first antibiotic effective against tuberculosis*
1954	John Franklin Enders, Thomas Huckle Weller, Frederick Chapman Robbins	*For their discovery of the ability of poliomyelitis viruses to grow in cultures of various types of tissue*
1958	Joshua Lederberg	*For his discoveries concerning genetic recombination and the organization of the genetic material of bacteria*
1960	Frank Macfarlane Burnet, Peter Brian Medawar	*For discovery of acquired immunological tolerance*
1965	François Jacob, André Lwoff, Jacques Monod	*For their discoveries concerning genetic control of enzyme and virus synthesis*
1966	Francis Peyton Rous	*For his discovery of tumour-inducing viruses*
1969	Max Delbrück, Alfred Day Hershey, Salvador Edward Luria	*For their discoveries concerning the replication mechanism and the genetic structure of viruses*
1972	Gerald M. Edelman, Rodney R. Porter	*For their discoveries concerning the chemical structure of antibodies*
1975	David Baltimore, Renato Dulbecco, Howard Martin Temin	*For their discoveries concerning the interaction between tumour viruses and the genetic material of the cell*
1976	Baruch S. Blumberg, D. Carleton Gajdusek	*For their discoveries concerning new mechanisms for the origin and dissemination of infectious diseases*
1980	Baruj Benacerraf, Jean Dausset, George D. Snell	*For their discoveries concerning genetically determined structures on the cell surface that regulate immunological reactions*
1984	Niels K. Jerne, Georges J.F. Köhler, César Milstein	*For theories concerning the specificity in development and control of the immune system and the discovery of the principle for production of monoclonal antibodies*
1987	Susumu Tonegawa	*For his discovery of the genetic principle for generation of antibody diversity*
1989	J. Michael Bishop, Harold E. Varmus	*For their discovery of the cellular origin of retroviral oncogenes*
1996	Peter C. Doherty, Rolf M. Zinkernagel	*For their discoveries concerning the specificity of the cell-mediated immune defence*
1997	Stanley B. Prusiner	*For his discovery of prions—a new biological principle of infection*
2005	Barry J. Marshall, J. Robin Warren	*For their discovery of the bacterium Helicobacter pylori and its role in gastritis and peptic ulcer disease*
2008	Harald zur Hausen	*For his discovery of human papilloma viruses causing cervical cancer*
2008	Françoise Barré-Sinoussi, Luc Montagnier	*For their discovery of the human immunodeficiency virus [HIV]*
2011	Bruce A. Beutler, Jules A. Hoffmann	*For their discoveries concerning the activation of innate immunity*
2011	Ralph M. Steinman	*For his discovery of the dendritic cell and its role in adaptive immunity*
2015	William C. Campbell, Satoshi Ōmura	*For their discoveries concerning a novel therapy against infections caused by roundworm parasites*
2015	Tu Youyou	*For her discoveries concerning a novel therapy against malaria*

While the spectrum of activity of most antibiotics is broad and fairly nonselective, monoclonal antibodies are usually highly specific in their effect and can provide an important basis for the development of effective vaccines. Monoclonal antibodies against bacteria such as *Staphylococcus aureus, Pseudomonas aeruginosa, Bacillus anthracis, Clostridioides difficile* and *Escherichia coli* are currently being explored. These antibodies are usually human antibody type IgG, whereby the respective complementarity determining regions (CDR) determine to which pathogenic bacterial antigens or strains the antibody binds [22]; however, this requires the identification of a reliable antigen structure in the pathogenic microorganism. Monoclonal antibodies can be produced from phage banks, B cell cultures, B cells immortalized by the Epstein-Barr virus (EBV) or by cloning single antigen-specific B cells [23]. Binding capacity and opsonophagocytic killing (OPK) are indicators of functional capacity and as such must be tested using a range of different test systems. If the putative antibodies show sufficient OPK, they can be tested in preclinical and clinical studies in animal models or humans to further evaluate their therapeutic potential.

The human monoclonal antibody 514G3, which is specifically directed against the cell wall protein *S. aureus*-specific staphylococcal protein A (SpA), has already been tested in both phase I and phase II studies. After initial determination of the maximum tolerated dose, the effect of the antibody in terms of safety and tolerance was investigated in patients suffering from *S. aureus *bacteremia, either methicillin-resistant or methicillin-susceptible *S. aureus* (MRSA or MSSA). For this purpose, a regimen consisting of antibody treatment in addition to standard antibiotic therapy was compared to standard antibiotic-only treatment (NCT02357966, https://clinicaltrials.gov). The results of the study are expected imminently. A high level of efficacy has already been shown in animal models. A survival rate of 60% was observed in experimental animals receiving the antibody prophylactically, compared to the control group in which no animal survived [24]. In another animal model, it was shown that monoclonal antibodies may also be useful in eradicating the colonization of the nasopharynx with MRSA [25]. There is also evidence that monoclonal antibodies are effective in biofilm-associated infections caused by *S. aureus *[23].

Another story of success in the quest for therapeutics and prophylactics to be used against antibiotic-resistant bacteria is constituted by the development of polyclonal and monoclonal antibodies, and the ongoing development of vaccines, against the bacterium *C. difficile*. Initially, whey from *C. difficile*-infected cows used in the treatment of *C. difficile*-associated diarrhea (CDI) in humans showed both efficacy with respect to recurrences of infection and good tolerability [26]. In the course of the study, toxin A and toxin B were identified as relevant immune target structures. The monoclonal antibody bezlotoxumab directed against toxin B in CDI is both efficacious and has the ability to reduce the recurrence rate of CDI by about 10% [27].

Based on the characterization of their neutralizing epitopes, three vaccines against *C. difficile* in humans have been established in phase II and phase III studies so far. While even formalin-inactivated toxins can still exhibit toxin-specific effects, immunogenic subunits of toxins, some of which are additionally genetically engineered, are better tolerated. A vaccine made by Sanofi Pasteur MSD that uses the toxoids A and B as antigen targets has been tested in a phase II study in Japan [28]. In addition, a genetically modified vaccine manufactured by Pfizer also using the toxoids A and B is currently being tested in a phase III study (NCT03090191, https://clinicaltrials.gov). A phase II study involving a recombinant fusion protein from toxins A and B (IC84/VLA84) manufactured by Valneva SE was recently completed (NCT01296386, https://clinicaltrials.gov) [29].

A completely different approach to immune modulation is the foreign stool transplantation or fecal microbiome transfer* (FMT)*. In this procedure, stool from carefully selected and tested donors is transplanted into the intestines of patients suffering from *C. difficile*. Although this form of bacteriotherapy is currently only being used in the context of (multiple) recurrent CDI, it has shown promising results [30]. The FMT has also been described to have positive results in the treatment of other diseases and in the eradication of colonization with antibiotic-resistant bacteria [31]; however, the long-term consequences and side effects of these complex interventions in the human microbiome have not yet been sufficiently explored. Careful donor selection and prior testing for infectious diseases and colonization with antibiotic-resistant microorganisms would seem to be particularly important. In fact, the FDA recently warned against the transfer of multidrug-resistant germs in the context of FMT [32].

Monoclonal antibodies, which are not directed against bacteria themselves but against individual components of the immune system, can also be beneficial in bacterial infections and serve as immune modulators. A classical example is eculizumab, a recombinant humanized antibody that binds specifically to the protein C5 in the complement system. This prevents its cleavage, which in turn prevents the formation of the membrane attack complex C5b‑9. Food-associated infections with *E. coli* serotype O157:H7 are characterized by shigatoxin-induced hemolytic uremic syndrome (HUS), which in turn is characterized by the triad of hemolytic anemia, thrombocytopenia and renal failure, accompanied by bloody diarrhea and neurological failures. In this process, shigatoxin binds complement factor H, resulting in an excessive response of alternative complement activation at the level of factor C3b. This overreaction is down-regulated by eculizumab, which binds factor C5 and can thus reduce cerebral damage in HUS [33]. As a consequence, however, infections with encapsulated bacteria (*N. meningitidis, H. influenzae, S. pneumoniae*) might occur more frequently, which is why appropriate vaccinations against encapsulated pathogens are highly recommended before using eculizumab [34].

In 1928, penicillin was discovered by the Scotsman Alexander Fleming, and on 12 February 1941 it was used for the first time as an antibiotic in a policeman suffering from staphylococcal osteomyelitis [35]. Another early antibiotic, developed by Gerhard Domagk in 1932, was sulfamidochrysoidin (Prontosil®), the first drug from the sulfonamide group [36]. At present, research into new antibiotics appears to be more important than ever, but also more difficult than ever since the challenge of antimicrobial resistance is expected to increase even further in the future. Normally, clinically valuable antibiotics are characterized by a broad spectrum of antimicrobial activity. Recently, with the discovery of ridinilazole, a narrow spectrum antibiotic has been developed that is comparatively specifically directed against *C. difficile*. The advantage of ridinilazole is that it minimizes the collateral changes to the microbiome that occur during antibiotic therapy with vancomycin or metronidazole, the current antibiotics of choice according to the guidelines. Although the mechanism of action has not yet been fully clarified, there is evidence that ridinilazole not only exerts influence on the bacteria itself in the gut, but also has therapeutic effects on the bacterial toxins A and B and additionally reduces the local inflammatory response [37, 38]. Phase III studies were initiated in 2019 (NCT03595566 and NCT03595553, https://clinicaltrials.gov) to further evaluate ridinilazole’s therapeutic value.

Nanoparticles and artificial liposomes can also be used in the treatment of bacterial infections as they are able to bind bacterial toxins and cause them to lose their toxin-specific effects. In mice, the administration of toxin-binding liposomes prevented the clinical consequences of sepsis caused by *S. aureus* or *S. pneumoniae* within 10 h after infection. Experimental animals which did not receive these liposomes died within 24–33 h [39].

Ever since the discovery of the human immunodeficiency viruses (HIV), people have been looking for effective ways to eliminate it in the body. Bi-specific T‑cell engagers (BiTE) are artificial monoclonal antibodies, which exhibit both a binding site for antigen structures and another for specific surface proteins on T cells. In vitro experiments have already shown that the viral replication of HIV is suppressed by inducing BiTE antibody-mediated redirection of T cells to gp120-expressing cells. It is currently being discussed whether the new generation of antibodies could be used to reach the replicative inactive viral reservoir of HIV-infected patients and thus achieve complete viral eradication. No therapeutic strategy to target the replicative inactive viral reservoir is available to date, although this reservoir is the main cause of viral rebound after the interruption of antiretroviral therapy. The reason for this is that the proviral DNA of HIV is anchored in the nucleus of host cells, meaning that replicative inactive cells are not recognized as infected cells by the host immune system [40]. A highly selective extraction of proviral DNA without damaging the host cell would be desirable. This goal seems to have been made feasible thanks to the clustered regularly interspaced short palindromic repeats (CRISPR) method [41], which has recently been used to successfully remove subgenomic HIV‑1 DNA fragments from the genome of HIV-infected mice. Up to 5 weeks after therapy, no virus was detectable in 5 of 13 animals, which suggests that the replicative inactive viral reservoir had been reached [42].

A Chinese scientist caused a scientific riot when he claimed that he had created the first genetically modified babies using the CRISPR method by inducing mutations, which ensured that the CCR5 receptor that serves as a major pathogenetic factor in early HIV spread, was not formed, thus protecting the newborns from infection with HIV [43]. At the moment it is unclear what further consequences this procedure will have for the babies. Such interventions in the genomes of unborn humans are ethically highly questionable and require a debate on the framework conditions for the use of the new technologies. In the CRISPR method, CRISPR RNA binds specifically to DNA sequences and marks relevant sites in the genome, which then are cut by the CRISPR-associated protein 9 (Cas9) nuclease. In this way, gene segments can be specifically cut out or inserted at certain sites. The same gene scissors can also be used to produce bacteriophages which can destroy specific gene segments or plasmids in bacteria and thus inhibit pathogenicity factors or selectively switch off specific resistance mechanisms [44]. Recently, a 15-year-old girl suffering from cystic fibrosis was successfully treated for disseminated resistant *Mycobacterium abscessus *infection after lung transplantation by means of selectively produced bacteriophages [45].

Another promising new method is the so-called T‑cell engineering in which specific receptors directed against non-self antigens of pathogenic microorganisms are inserted into the body’s own T‑lymphocytes to give T cells the new ability to target a specific protein. After in vitro modification, the altered lymphocytes are returned to the patient. Currently, this method is mainly used in the field of oncology; however, a T-cell-based therapy against pathogenic microorganisms also seems conceivable and offers hope for curative responses in patients with drug-resistant infectious disease [46].

New technical possibilities continue to permit the development of interesting and effective therapeutics and vaccines. Particularly desirable next milestones in this context would be not only vaccines against HIV and tuberculosis, but also cross-species vaccines against various multidrug-resistant bacterial pathogens.
